# Fast Method of Registration for 3D RGB Point Cloud with Improved Four Initial Point Pairs Algorithm

**DOI:** 10.3390/s20010138

**Published:** 2019-12-24

**Authors:** Peng Li, Ruisheng Wang, Yanxia Wang, Ge Gao

**Affiliations:** 1 School of Geographic Information and Tourism, Chuzhou University, No. 1 Huifeng West Road, Chuzhou 239000, China; lipeng@chzu.edu.cn (P.L.); surveymapping@126.com (Y.W.); cicga635457049@163.com (G.G.); 2 School of Geographical Sciences, Guangzhou University, No. 230, Waihuan West Road, Guangzhou 510006, China; 3 Department of Geomatics Engineering, University of Calgary, 2500 University Drive NW, Calgary, AB T2N 1N4, Canada

**Keywords:** 3D point cloud, point cloud registration, global registration, RGB, four initial point pairs, RGB-FIPP

## Abstract

Three-dimensional (3D) point cloud registration is an important step in three-dimensional (3D) model reconstruction or 3D mapping. Currently, there are many methods for point cloud registration, but these methods are not able to simultaneously solve the problem of both efficiency and precision. We propose a fast method of global registration, which is based on RGB (Red, Green, Blue) value by using the four initial point pairs (FIPP) algorithm. First, the number of different RGB values of points in a dataset are counted and the colors in the target dataset having too few points are discarded by using a color filter. A candidate point set in the source dataset are then generated by comparing the similarity of colors between two datasets with color tolerance, and four point pairs are searched from the two datasets by using an improved FIPP algorithm. Finally, a rigid transformation matrix of global registration is calculated with total least square (TLS) and local registration with the iterative closest point (ICP) algorithm. The proposed method (RGB-FIPP) has been validated with two types of data, and the results show that it can effectively improve the speed of 3D point cloud registration while maintaining high accuracy. The method is suitable for points with RGB values.

## 1. Introduction

Three-dimensional (3D) point cloud registration is very important in 3D point cloud data processing, which can provide support for post-processing such as feature extraction, 3D modeling, object recognition, etc. Because three-dimensional coordinate values of each point cloud data are acquired with a scanner as the coordinate origin, the data collected at each station must be stitched together into the same scene through point cloud registration. Given two point cloud datasets, *P* is the target point cloud and *Q* the source point cloud. Point cloud registration is the process of looking for an optimal rigid transformation matrix so that source point cloud *Q* can be transformed into *Q*’ and the overlapping regions of *P* and *Q*’ are as close as possible.

In order to solve this question, Besl and McKay [1] proposed the iterative closest point (ICP) algorithm in 1992. Based on iterative optimization, the ICP algorithm solves the transformation matrix by using pairs of nearest 3D points in the target and source datasets as correspondences, and transforms the original point dataset into new coordinates by using the matrix. It then repeats the above steps until the accuracy requirements are satisfied. The ICP algorithm can achieve high registration accuracy, and it is very robust. However, it has drawbacks, such as being susceptible to local minima, having a small convergence basin, and generally requiring a high number of iteration steps until convergence can be reached [2]. To solve this problem, many researchers have conducted studies to improve the ICP algorithm, which include improving the method of resampling [3], devising a better way to search for point-to-point correspondences [4], speeding up registration based on a dynamic adjustment factor [5], and improving its registration accuracy [6]. However, there has no single method that can solve all these problems with the ICP algorithm, although there are many ways to improve it.

Another approach to improve registration involves dividing the processing of 3D registration into two stages: global registration (or coarse registration) and local registration (or fine registration). In the stage of global registration, the two point cloud datasets *P* and *Q* are roughly aligned together, and, in the second stage, a local registration based on the ICP algorithm is performed. Because after global registration the locations of two datasets are very close, this method can avoid the disadvantages of ICP, accelerate the convergence rate, and reduce the number of iterations when performing local registration.

Research into global registration is concentrated on three aspects: descriptors, searching strategies, and the rigid transformation matrix. First, descriptors aim at encoding the local shape around a point in terms of a set of numerical values. By using descriptors, the differences between every point can be calculated, and according to a criterion a small number of points with significant characteristics can be chosen. This can not only reduce the computation time and the number of points to be considered, but can also find the similarity of the same points in two point cloud datasets. In these descriptors, normal and surface curvature [7] are not robust and easily affected by noise and neighbor radius, moment invariants [8], and spherical harmonic invariants [9], and integral volume descriptors [10] are robust, but still sensitive to noise. The final approach is to apply object recognition to point cloud registration, e.g., spin image (SI) [11], 3D shape context (3DSC) [12], point signatures [13], curvature-based histograms [14], a point feature histogram (PFH) [15], a fast point feature histogram (FPFH) [15], rotational projection statistics (RoPS) [16], a local surface patch (LSP) [17], and tri-spin-images (TriSI) [18], signatures of geometric centroids (SGC) [19], local voxelized structure (LoVS) [20], triple orthogonal local depth images (TOLDI) [21], rotational contour signatures (RCS) [22], and rotational silhouette maps (RSM) [23]. However, the computational complexity of calculating descriptors is *O*(*kn*) at best, such as in a FPFH [19], where *k* is the number of neighborhood points to a given point and *n* is the number of points in a dataset [24].

Second, searching strategies are used to find the proper point-to-point correspondences between two datasets. Theoretically, at least three points pairs in two datasets are needed to determine a rigid transformation matrix. If all points in two datasets need to be searched, the cost is *O*(*n*^6^). For the requirement of fast speed in global registration, this is inadvisable. There have two methods advanced to solve this problem. The first method extracts a small number of points in order to reduce the size of *n*, which still belongs to the step of calculating the descriptor. The second is to improve the searching strategy so that the computational complexity can be reduced. Presently, the more mature methods are random sample and consensus (RANSAC) [25], random sampling (RANSAM) [26], four-points congruent sets (4PCS) [27], greedy initial alignment (GIA) [14], four initial point pairs (FIPP) [28], and evolutionary computation (EC) [29]. Among these, the computational complexity of GIA and FIPP are, respectively, *O*($C^{2\cdot }{}^{\ln2^{n}}$) and *O*(*kC*^4^), which are both less than that of the other methods, where *k* and *C* are the number of neighborhood points and candidate points, respectively.

After determining the searching strategies, point-to-point correspondences between two datasets can be determined and, finally, a rigid transformation matrix involving a three-dimensional coordinate transformation will be solved. The traditional method of 3D coordinate transformation is least squares (LS) [30]. In addition, the methods of total least squares (TLS) [31,32], weighted total least squares (WTLS) [33,34,35], and robust weight total least squares (RWTLS) [36,37] were proposed to solve problems such as the coefficient matrix, gross error, etc. In 3D point cloud registration, point-to-point correspondences are determined by distances between points, so there can be no gross errors in points for registration because of the constraint of distances. In this paper, TLS is used to solve the rigid transformation matrix.

Overall, the conventional steps of point cloud registration are global registration and local registration. Global registration includes calculating descriptors, searching point-to-point correspondences and solving a rigid transformation matrix. Local registration usually performs the ICP algorithm. The purpose of global registration is to roughly align the two datasets together, so as not to make the ICP fall into a local optimum during subsequent local registrations. Therefore, global registration focuses more on speed than accuracy, because the final accuracy of registration is determined by the results of local registration and the ICP algorithm has been proven to have a high accuracy. In this paper, we focus on finding a fast method of global registration to improve the efficiency of point cloud registration.

Color is the important information in a point cloud that can be obtained directly from the point cloud dataset. Color information usually consists of three channels: red, green, and blue (referred to as RGB). RGB value is usually expressed in two ways, i.e., integer and floating-point. In the integer expression, the values of each channel range from 0 to 255; in floating-point, the three channels are merged into 24-bit binary numbers. When two point clouds with color information are used for registration, RGB values of the same points in the overlapping area are the same or similar in theory. This provides the possibility of searching point-to-point correspondence. At the same time, RGB values for each point are independent, and we do not need to consider the relationship between a point and its neighbors. Therefore, if RGB value is used to describe the feature of a point instead of descriptors, the computational complexity of extracting feature points will be reduced from *kn* to *n*, which will greatly improve the registration efficiency. At present, there are few studies on point cloud registration based on RGB values. Johnson [38], Druon [39], Hao [40], and Ren [41] used point cloud color information for the ICP algorithm to accelerate the registration, but the RGB value is only an additional condition, and the drawbacks of the ICP algorithm have yet to be solved. Yamashita [40] used RGB value to solve the problem of underwater image registration, but the images were 2D.

In this paper, we propose a fast method of registration based on RGB value of a point and an improved FIPP algorithm (RGB-FIPP). The RGB-FIPP algorithm performs global registration by using the RGB value of a point as a descriptor, the improved FIPP algorithm as the search strategy of point-to-point correspondences, and the ICP algorithm for local registration. The results show that the proposed method is faster and has high accuracy.

## 2. Searching Strategy with FIPP Algorithm

This section may be divided by subheadings. It should provide a concise and precise description of the experimental results, their interpretation as well as the experimental conclusions that can be drawn. The FIPP algorithm [28] is mainly used in point cloud registration. It searches the correspondences of the point pairs for registration from two point cloud datasets within three constraints: features of points, distances between points, and location relationships. Features of points is used to generate the candidate point set for a given point, distances between points can match the same point pairs from two point cloud datasets, and location relationships are used to prevent the overall point pairs from being in the wrong direction.

Given two point cloud datasets *P* and *Q*, where *P* is the target dataset and *Q* is the source dataset, FIPP first chooses *l* points from *P* that are evenly distributed over the dataset and then randomly selects four points from *l*. The purpose of this is to ensure that those four points are in the overlap area. Second, candidate point set *C* in *Q* of four points in *P* are generated, and all combinations of the candidate point set are traversed for each of the four points. The combination, which has the smallest difference between the distance and consistent direction, comprises the corresponding points of the four points. If all differences between the distances in candidate points are more than threshold or if direction is inconsistent, the current four points in *l* are discarded and new points are reselected until four initial point pairs are determined. Next, based on the four initial point pairs, new points *p_i__+j_* from *P* are added in turn, and the best candidate points *q_i_*_+*j*_ are also chosen within the constraints of feature and distance, until the number of point pairs satisfies point cloud registration. After that, the final point pairs can be used to generate a rigid transformation matrix.

The reason that the number of initial point pairs is four is that the new point satisfying the distance constraint is unique and the best candidate point is determined only by the constraints of feature and distance, not location relationship.

**Figure 1:**
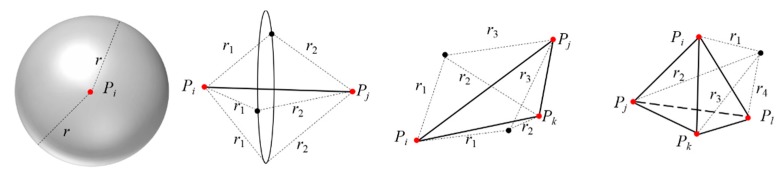
The possibility of new points with different number of initial points, the number respectively are 1,2,3,4 from left to right.

As can be seen from Figure 1, if the number of initial points is 1, the new points satisfying the distance constraint are likely to be located in any position on a sphere, where the distance between the new points and *p_i_* is *r*. Likewise, the new points may be a circle or two points, respectively, if the number is 2 or 3. Only when the number is 4 is the new point unique.

Meanwhile, four initial points from *P* must be in the overlap area; otherwise, corresponding points from *Q* cannot be found or the point-to-point correspondences are wrong. To ensure this, *l* points selected from *P* are as evenly distributed as possible by setting a minimum distance *d* between any two points, so the distance between any two points in the *l* points satisfies the following formula:

(1) $$\sqrt{{\left(x_{p_{i}}-x_{p_{j}}\right)}^{2}+{\left(y_{p_{i}}-y_{p_{j}}\right)}^{2}+{\left(z_{p_{i}}-z_{p_{j}}\right)}^{2}}>d$$

These *l* points may not all be in the overlap area, but the probability of four of which being greater and *l* points being distributed evenly also increases this probability.

**Figure 2:**
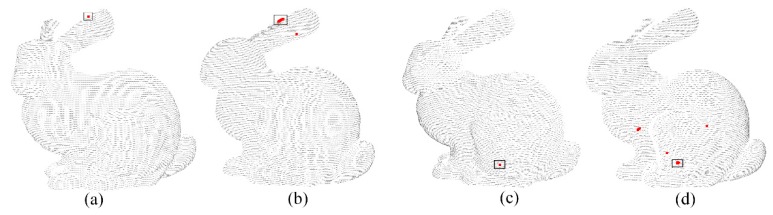
Candidate points in *P* of two different feature points in *Q*: (**a**) A feature point of source dataset (**b**) The candidate points of target dataset corresponding to point of a (**c**) Another feature point of source dataset (**d**) The candidate points of target dataset corresponding to point of c.

Once four initial points in *P* are chosen, the key of the FIPP algorithm is the searching strategy for the corresponding points of four initial points. At first, four corresponding candidate point sets for four initial points from source dataset *Q* are constructed as follows:

(2) $$C=\left\{c_{i}|c_{i}=\langle p_{i},q_{i1},q_{i2},q_{i3},\cdots ,q_{ik},1\leq i\leq 4\rangle \right\}$$

where *k* is the number of candidate points for *p_i_*. The candidate points for each point are selected by calculating the feature similarity of all points. Only *k* points with the most similar characteristics are regarded as the candidate points, which can improve the search efficiency of the corresponding points. Figure 2 shows the results of selecting candidate points, where Figure 2b,d are candidate points of Figure 2a,c, respectively.

The distances between any two points from four initial points in *P* are then calculated; meanwhile, all combinations of candidate point set in *Q* are traversed. Finally, the candidate point combination with the minimum distance differences corresponding to four points is considered to be the best corresponding points, and the formula is as follows:

(3) $$\sqrt{{\left(x_{p_{i}}-x_{p_{j}}\right)}^{2}+{\left(y_{p_{i}}-y_{p_{j}}\right)}^{2}+{\left(z_{p_{i}}-z_{p_{j}}\right)}^{2}}-\sqrt{{\left(x_{q_{ir}}-x_{q_{jr}}\right)}^{2}+{\left(y_{q_{ir}}-y_{q_{jr}}\right)}^{2}+{\left(z_{q_{ir}}-z_{q_{jr}}\right)}^{2}}<\sigma_{d},$$

where *σ_d_* is the threshold for the difference of distance. If all combinations of candidate points cannot satisfy Equation (3), four initial points are discarded, and four new points will be reselected from *l* points.

Additionally, another constraint describing the relative position relationship between points is used to ensure that the relative orientation of points is consistent, and it is expressed as

(4) $$q_{ir}=\{\begin{array}{l} true,\left(z_{p_{i}}>z_{p_{j}}\cap z_{q_{ir}}>z_{q_{jr}}\right)\cup \left(z_{p_{i}}<z_{p_{j}}\cap z_{q_{ir}}<z_{q_{jr}}\right)\\ false,\left(z_{p_{i}}>z_{p_{j}}\cap z_{q_{ir}}<z_{q_{jr}}\right)\cup \left(z_{p_{i}}>z_{p_{j}}\cap z_{q_{ir}}<z_{q_{jr}}\right) \end{array}.$$

**Figure 3:**
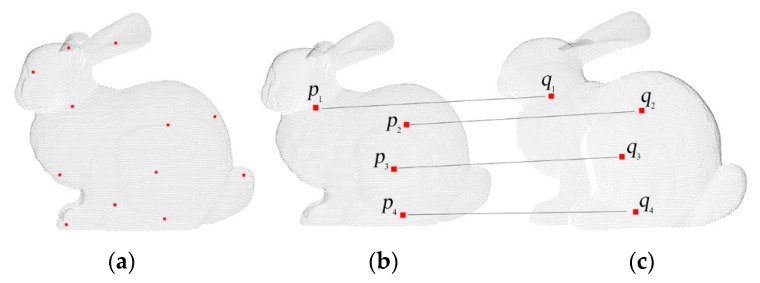
The process of Initial four point pairs by using FIPP algorithm: (**a**) Evenly distributed *l* points in target dataset (**b**) Initial four points from *l* in target dataset (**c**) Four corresponding points in source dataset.

As a rule, when a 3D scanner scans the object, the instrument is perpendicular to the ground, so the *z* values of the same two points in both point datasets have a consistent relationship; that is, if the *z* value of *p_i_* is greater than *p_j_* in *P*, then *q_i_* must also be greater than *q_j_* in *Q*. The FIPP algorithm determines the four initial point pairs by traversing combinations of candidate points within the constraints of feature similarity, the same distance, and direction consistency. Figure 3 is the process of selecting four point pairs.

After four initial point pairs are selected, new point pairs are selected in turn. New point *p_i+j_* in *P* is constrained by Equation (1), while *q_i+j_* is constrained by Equations (2) and (3), but not Equation (4), because the distance between the new point and four initial points are unique.

## 3. Candidate Point Set Based on RGB Value

The FIPP algorithm has been used in point cloud registration without RGB value [28], which used a point descriptor as the feature constraint. The results show that the accuracy of the FIPP algorithm with a descriptor is good in five types of point cloud datasets. However, a point feature descriptor such as a FPFH must calculate the multi-dimensional histogram of all points by using their neighborhood points, so the computational complexity is *O*(*kn*) in the best of situations, and some are even *O*(*k*^2^*n*), where *k* is the number of neighborhood points and *n* is the number of points in a dataset. In practice, the time for calculating descriptors will be longer because the result is not a single value, but a multi- or high-dimensional histogram. In this paper, we propose a faster method of registration called RGB-FIPP, which uses the RGB value as a point feature to generate candidate points and uses an improved FIPP algorithm to search correspondences.

### 3.1. Statistics of Points of the Same Color

RGB value is the most intuitive and simplest feature in a point cloud dataset. Generally, the same points in different datasets should have the same or similar color, so the search range of the corresponding points can be reduced by looking for points with the same or similar color from two datasets. In other words, attention is shifted away from a large number of points towards the different colors, which is similar to feature point extraction.

**Figure 4:**
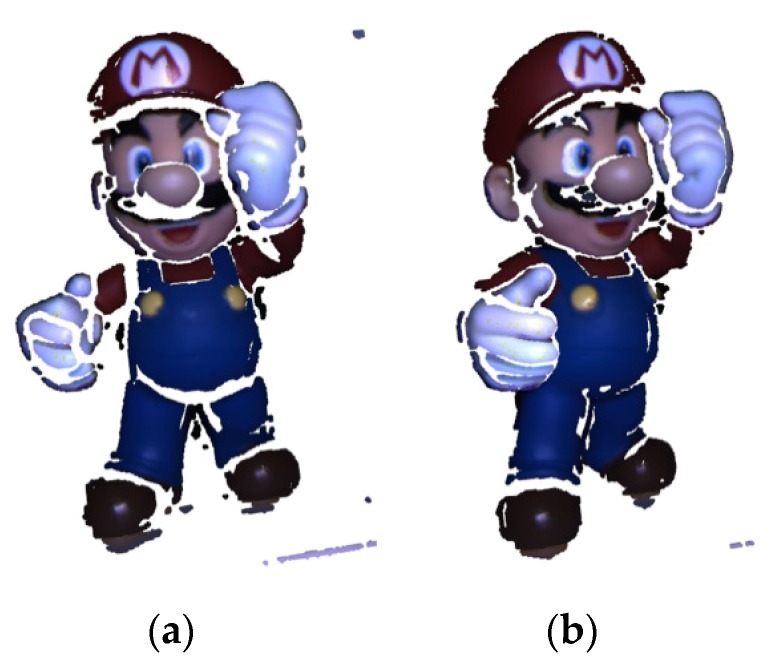
Point cloud datasets with color: (**a**) Target dataset (**b**) Source dataset.

**Figure 5:**
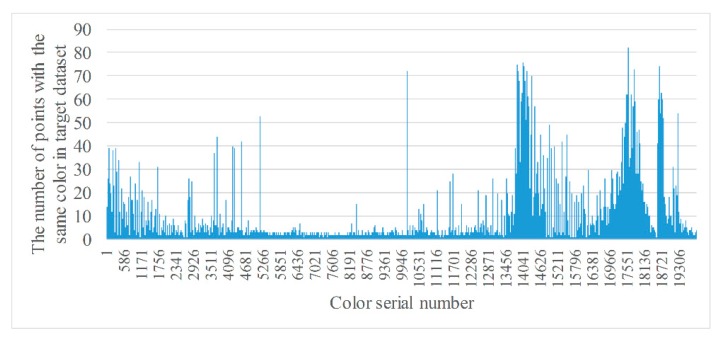
Statistics of points with the same color from target dataset.

**Figure 6:**
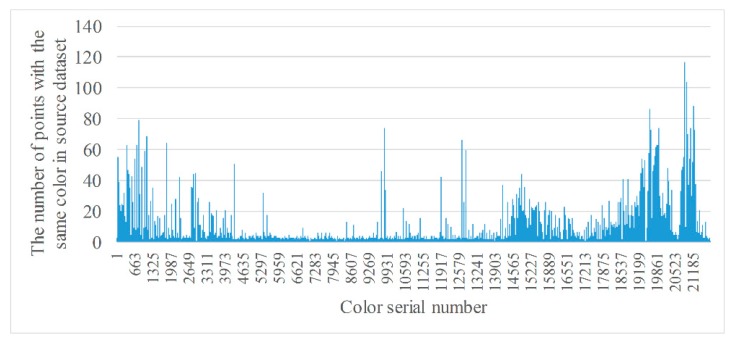
Statistics of points with the same color from source dataset.

Figure 4 shows two RGB point cloud datasets. The left-hand figure is a target dataset with 45,205 RGB points and the right-hand figure a source dataset with 53,949 RGB points. Because every point has a RGB value, some points in a dataset may have the same color. We can count the number of colors and classify points with the same color into the same class. Figure 5 and Figure 6 show, respectively, the number of different colors from the target dataset and source dataset.

It can be seen from Figure 5 and Figure 6 that, there are approximately 20,000 colors in the target and source, datasets, respectively, and the number of points of the same color ranges from 1 to over 100. However, the number of colors is still high even though it has decreased by more than half. In addition, colors with a smaller number of points are much more numerous than those with a large number of points. Therefore, the distribution of colors should be counted in order to understand the proportion of colors with the same number of points.

Table 1 shows the distribution of colors with the same number of points in two datasets. As a general rule, colors with one point account for nearly 80% (79.34% and 78.38%) of all points, two points account for nearly 10% (9.79% and 10.40%), and the more points with the same color, the smaller the proportion of color. However, if the number of points of the same color is too few, these points are probably not the same points in the two datasets, so they have little effect on registration.

### 3.2. Color Filtering

**Figure 7:**
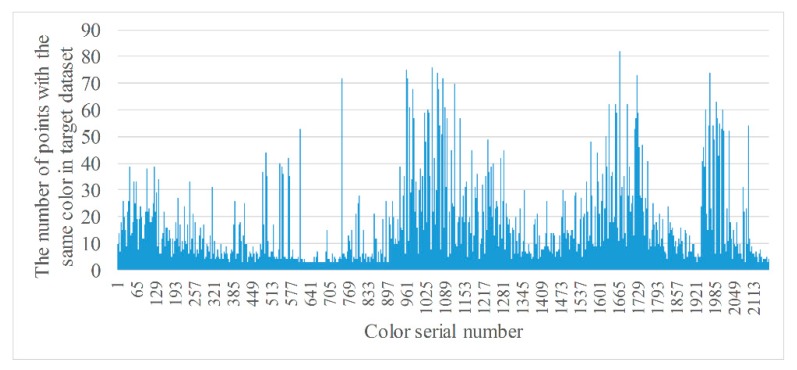
Statistics of points with the same color from target dataset after filtering.

**Figure 8:**
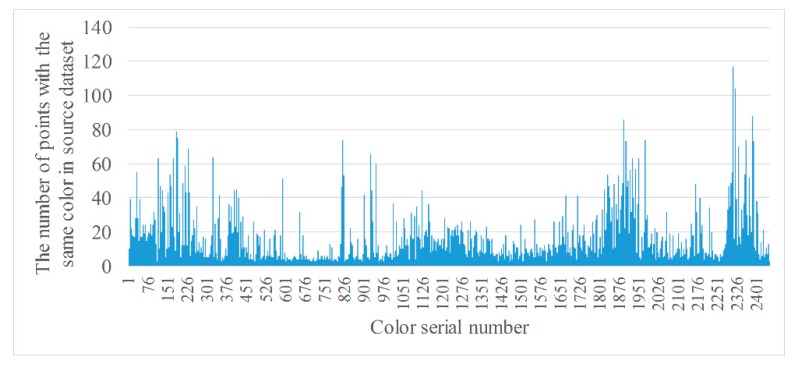
Statistics of points with the same color from source dataset after filtering.

The FIPP algorithm must search point-to-point correspondences within the constraint of feature, and now that constraint will be replaced by RGB value. With these two motivations, colors with a small number of points must be filtered by setting a threshold, and the formula is

(5) $$RGB_{i}=\{\begin{array}{l} true,\;N_{RGB_{i}}>\sigma_{n}\\ false,\;N_{RGB_{i}}\leq \sigma_{n} \end{array}$$

where *RGB_i_* is the RGB value of the ith color, $N_{RGB_{i}}$ the number of points for which the RGB value is *RGB_i_* in a point cloud dataset, and *σ_n_* the threshold used to remove color with a small number of points. If $N_{RGB_{i}}$ is greater than *σ_n_*, the color is retained, as well as all points with that color, and vice versa. The purpose of this is to reduce the number considered when searching point-to-point correspondences in the two datasets. Figure 7 and Figure 8 are the statistics of points with the same color from two datasets after filtering.

From Figure 7 and Figure 8, it can be seen that the number of types of colors are greatly reduced after filtering, to 2162 and 2450, respectively.

### 3.3. Points of the Same Color from Two Datasets

**Figure 9:**
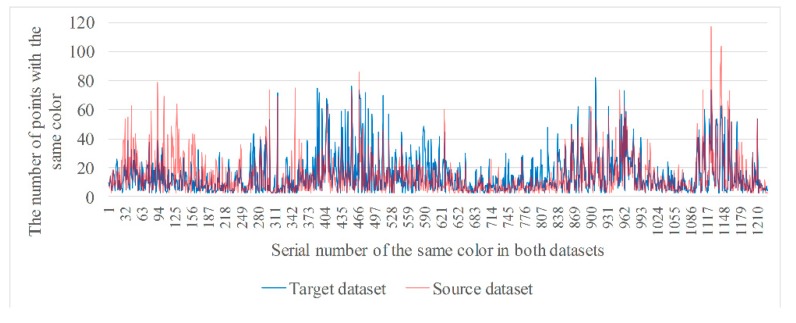
Comparison of the points with the same color from two datasets.

Based on the discussion in the preceding subsection, the corresponding points of the same color from two datasets can be confirmed, and the formula is

(6) $$\{\begin{array}{l} p_{i}=true\cap q_{j}=true,\;RGB_{p_{i}}=RGB_{q_{j}}\\ p_{i}=false,\;RGB_{p_{i}}\neq \left\{RGB_{{}_{q}}|q=1,2\cdots m\right\}\\ q_{j}=false,\;RGB_{q_{j}}\neq \left\{RGB_{p}|p=1,2\cdots n\right\} \end{array}$$

where *p_i_* and *q_j_* are, respectively, the *i*th and *j*th points in the target and source datasets, $RGB_{p_{i}}$ and $RGB_{q_{j}}$ are the RGB values of points *p_i_* and *q_j_*, respectively, *RGB_p_* and *RGB_q_* are the values of the *p*th and *q*th colors in the two datasets, respectively, and *m* and *n* are the number of types of colors in the two datasets. Only when the RGB values of points *p_i_* and *q_j_* are equal can point-to-point correspondences be built. Figure 9 shows a comparison of the points from two datasets with the same color correspondence.

In Figure 9, the blue lines indicate the number of points of the same color in the target dataset and the red lines indicate that of the source dataset. In terms of the number of colors, it is decreased by approximately half because some colors in the two datasets are not the same. In terms of the number of points of the same color, there are large differences in the same color from the two datasets. In some colors, the number of points in the target dataset is far greater than in the source dataset, while being far less in others. The reason for this is that the two datasets were acquired twice and there was a slight deviation in color between the two images.

### 3.4. Candidate Points Set with RGB Value Tolerance

**Figure 10:**
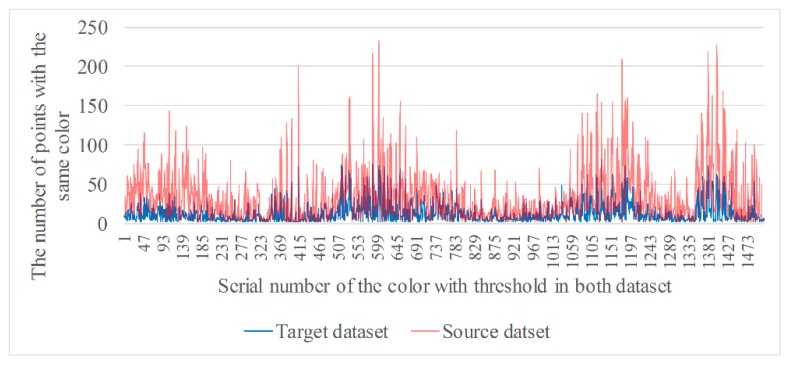
The comparison of points with the same color from two datasets by setting threshold.

However, point-to-point correspondence is very critical in FIPP. It may obtain the incorrect result if the number of points of the same color in both the target and source datasets varies widely. Meanwhile, point-to-point correspondences in the two datasets are searched with FIPP by considering points from the source dataset as the candidate set of points of the same color from the target dataset. Thus, if the number of candidate points is too small, it is very difficult to determine the point-to-point correspondences. One solution is to set a color tolerance to points in the source dataset, while leaving the points in the target dataset unchanged. In this way, the number of candidate points is increased to improve the possibility of point-to-point correspondence. The formulas are

(7) $$RGB_{q_{i}}\{\begin{array}{l} \in RGB_{j},\left|RGB_{q_{i}}-RGB_{j}\right|\leq \sigma_{c}\\ \notin RGB_{j},\left|RGB_{q_{i}}-RGB_{j}\right|>\sigma_{c} \end{array}$$

(8) $$RGB_{q_{i}}\{\begin{array}{c} \in RGB_{j},\left|R_{q_{i}}-R_{j}\right|\leq \sigma \cap \left|G_{q_{i}}-G_{j}\right|\leq \sigma \cap \left|B_{q_{i}}-B_{j}\right|\leq \sigma_{c}\\ \notin RGB_{j},\left|R_{q_{i}}-R_{j}\right|>\sigma \cup \left|G_{q_{i}}-G_{j}\right|>\sigma \cup \left|B_{q_{i}}-B_{j}\right|>\sigma_{c} \end{array}$$

where Formulas (7) and (8) are applied to the case in which the values of color belong to the types of floating-point and RGB integers, respectively. $RGB_{q_{i}}$ is the color value of point *q_i_* in the source dataset, *RGB_j_* the *j*th color in the source dataset, *σ_c_* the threshold for color tolerance, and $R_{q_{i}}$, $G_{q_{i}}$, and $B_{q_{i}}$ are the values of R, G, and B, respectively, of point *q_i_*. When the type of color is floating-point, the point *q_i_* belongs to the *j*th color if the absolute value of the difference between $RGB_{q_{i}}$ and *RGB_j_* is less than *σ_c_*, and vice versa. Similarly, when the type of color is integer with a *R*, *G*, and *B* value, the point *q_i_* belongs to the *j*th color if all absolute values are less than *σ_c_*. Figure 10 shows a comparison of the points from two datasets with the same color correspondence using color tolerance.

**Figure 11:**
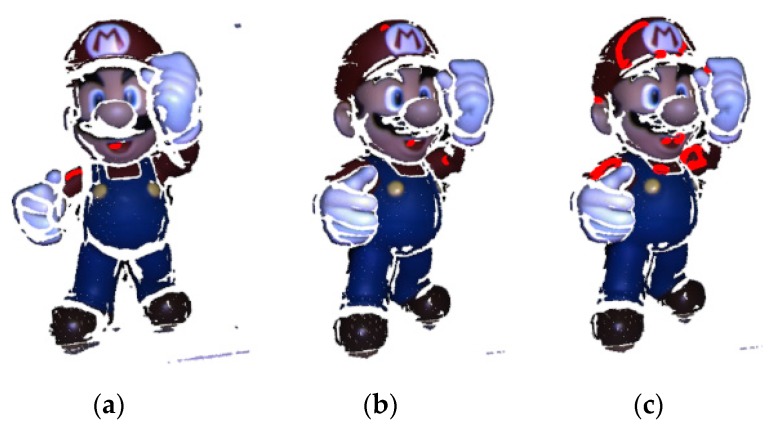
The different results of candidate points extraction before and after using color tolerance: (**a**) Points with a given color from target dataset (**b**) Candidate points with the same color from source dataset before using color tolerance (**c**) Candidate points with the same color from source dataset after using color tolerance.

The types of colors in Figure 10 are slightly more in number than those in Figure 9. This is because corresponding points in color for some points in the target dataset, which have none of the same colors as in the source dataset, can be found by setting the threshold for color tolerance. Additionally, the number of points with the same color in the source dataset has been increased, while the number in the target dataset remains the same. In this way, the possibility that a point in the target dataset can be searched for the corresponding point from the source dataset is increased. In order to apply RGB value to the FIPP algorithm to implement point cloud registration, the formula of target points and candidate points set with the same color is improved as follows:

(9) $$\{\begin{array}{l} C_{p}=\left\{c_{p_{i}}|c_{p_{i}}=\langle RGB_{i},p_{i1},p_{i2},p_{i3},\cdots ,p_{ik},1\leq k\leq m\rangle \right\}\\ C_{q}=\left\{c_{q_{i}}|c_{q_{i}}=\langle RGB_{i},q_{i1},q_{i2},q_{i3},\cdots ,q_{il},1\leq l\leq n\rangle \right\} \end{array}$$

where *C_p_* and *C_q_* are point sets with the same color in the target and source datasets, $c_{p_{i}}$ and $c_{q_{i}}$ are point sets both with *i*th color, *p_ik_* and *q_il_* are the *k*th and *l*th points in their respective set, respectively, and *k* and *l* are the number of points in each respective set. Different from the situation of only one point from the target dataset and several points from the source dataset in the candidate point set, where the descriptor is regarded as the feature of a point, the candidate points sets both consist of more than one point from the two datasets when RGB value serves as the feature of a point. Figure 11 represents the different candidate point sets before and after using color tolerance.

In Figure 11b, the points in the target dataset and the candidate point of the same color are both displayed in red. It can be seen that there are few points in the same location without color tolerance between the two datasets, and after using color tolerance the number of candidate points increases as do the same points. Furthermore, when searching point-to-point correspondence using the FIPP algorithm, the possibility of success in finding point pairs becomes higher with more candidate points. Nevertheless, more candidate points may increase the time to search point pairs because FIPP is implemented by traversing all combinations of candidate points. Therefore, it is very important to choose a reasonable threshold for color tolerance.

Unlike descriptors, RGB value regarded as the feature of point is obtained without considering neighborhood points of each point; therefore, the computational complexity is *O*(*n*) in the stage of building the candidate point set, which is far less than that for descriptors. It is worth mentioning that, when using FIPP to search point-to-point correspondence, time of RGB will be greater than that of descriptors because most descriptors represented by high-dimensional histograms that can distinguish subtle differences of points result in fewer candidate points. Therefore, it is very critical to set the proper threshold *σ_c_* in Formulas (7) and (8). In Section 5, we will discuss the influence of *σ_c_* on registration.

## 4. RGB Point Cloud Registration

**Figure 12:**
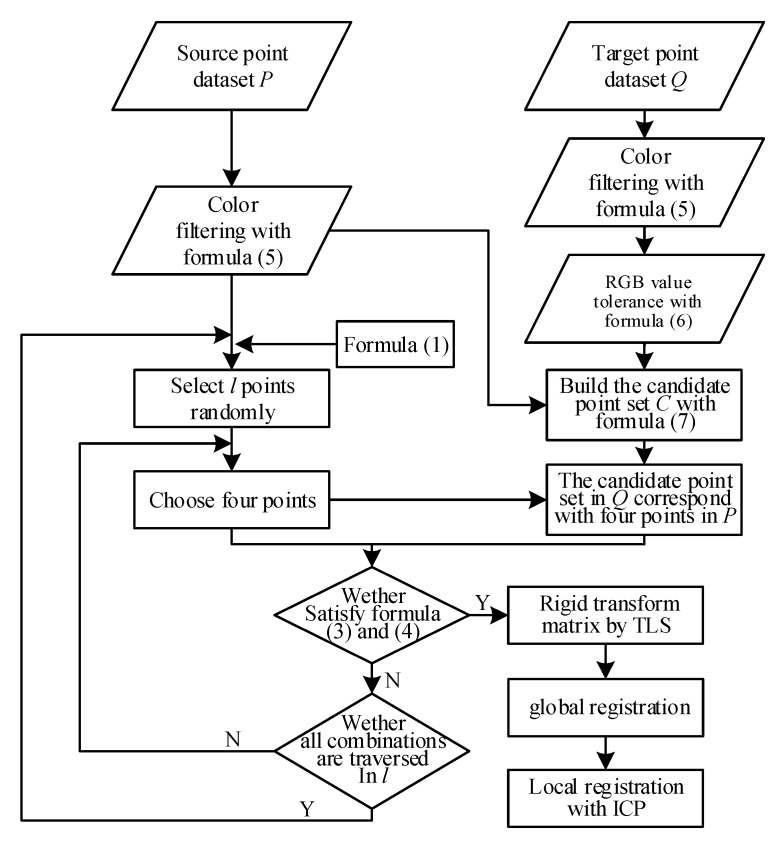
3D RGB point cloud registration flow chart.

When a candidate points set with color tolerance is defined, the FIPP algorithm can be performed to look for point-to-point correspondence between two point cloud datasets. After this, a rigid transformation matrix can be obtained with point-to-point correspondence as to global registration, and then point cloud registration is accomplished by local registration. In this section, the FIPP algorithm is improved for searching strategy with RGB value, a rigid transformation matrix of global registration is calculated TLS, and local registration is calculated by the ICP algorithm. The complete 3D point cloud registration flow chart is presented in Figure 12.

### 4.1. Improvement of FIPP Algorithm in 3D RGB Point Cloud Registration

When the FIPP Algorithm is applied to point cloud registration by using FPFH, candidate points are generated with the similarity of FPFH, and then point-to-point correspondences are searched. FPFH is a high-dimensional descriptor, so it is able to distinguish subtle differences between points from a point cloud dataset. RGB value is only a number that has a low degree of differentiation, so registration accuracy with RGB value is lower than with FPFH. However, RGB value only applies to global registration, which focuses more on efficiency, and the accuracy can be guaranteed in the local registration. In FIPP, four initial point pairs are searched at first, and on this basis new point pairs are constantly being generated until the number is satisfied. The purpose of adding new point pairs is to improve the accuracy. However, if RGB value is used in FIPP, new point pairs do not make any contribution to the improvement of registration accuracy.

A new point pair is verified by calculating three constraints and the total computational complexity of new point pairs is *k*·*n_l_*·*n_c_*, where *k* denotes the number times of successfully finding a correct point in *P* that have the same point in *Q*, *n_l_* is the number of point pairs that needs to be added, and *n_c_* is the number of candidate points. When FPFH is used in FIPP, *k* is small because FPFH has a high degree of differentiation on points, and it is highly probable that the candidate points in *Q* of a given point in *P* contain the same point, so the time for searching new point pairs is not very long. While RGB value is used in FIPP, *k* may be greatly increased as well as search time because of the low degree of differentiation in RGB.

From the above discussion, new point pairs not only cannot improve registration accuracy, but can also increase runtime; meanwhile, in global registration, runtime and efficiency are of greater concern. In this way, new point pairs are not necessary, and in this paper the FIPP algorithm is simplified to only search for four initial point pairs in order to improve efficiency.

### 4.2. Rigid Transformation Matrix of Global Registration

After four initial point pairs are determined, a rigid transformation matrix can be calculated with these point pairs for global registration. There are many ways to calculate a rigid transformation matrix, mainly including LS, TLS, WTLS, and RWTLS. In this paper, point pairs for local registration are chosen by FIPP, which are determined by the distances between points, so there is no gross error in point pairs. Therefore, TLS can solve the problem. Because the algorithm of TLS is very mature, the principle of TLS is not described in this paper.

### 4.3. Local Registration with ICP

Two point cloud datasets are roughly aligned together after global registration; however, local registration is also required in order to align two datasets accurately. In this paper, local registration is performed with the ICP algorithm, which aligns two datasets accurately through iteration; the steps are as follows:

1. Search the nearest point in target dataset *P* for every point in source dataset *Q*, and constitute point-to-point correspondence;
2. Generate a rigid transformation matrix by using point-to-point correspondence, and transform the source dataset into a new dataset with the matrix;
3. Determine whether the value of *RMS*(P*,Q*) is less than a threshold (see Equation (10)); if greater, go back to step 1 and continue; otherwise, proceed to the end:

(10) $$RMS(P^{*},Q^{*})=\sqrt{\frac{1}{n}\sum_{i=1}^{n}{\left(\left\|p_{i}-q_{j}\right\|\right)}^{2}}<\sigma ,(1\leq j\leq m)$$

where *p_i_* is the nearest point in the target dataset of *q_j_* in the source dataset, *m* and *n* are the number of *P* and *Q*, respectively, and *σ* is the threshold for minimum distance between the two datasets.

Despite the ICP algorithm having some drawbacks, such as local minima and high computational complexity, etc., it can eliminate defects and achieve a high precision of registration after global registration.

### 4.4. Evaluations of Registration Results

Evaluations of registration results include two aspects: efficiency and accuracy. In terms of efficiency, runtime is an important indicator. By analyzing the different runtimes with different parameters, the law of efficiency can be revealed. In addition, by comparing runtimes between RGB-FIPP and FIPP, the advantages of the algorithm’s efficiency can be verified. By running the RGB-FIPP algorithm continuously several times, its stability can be tested. In addition, the entire runtime with RGB-FIPP consists of several parts: generating candidate points with RGB value, searching point-to-point correspondences with improved FIPP, and performing local registration with ICP. Runtime for each of these parts will be determined separately in order to determine the specific advantages of the algorithm.

Regarding accuracy, the method of using RGB value to find point-to-point correspondences is less accurate than FIPP because RGB is only a value, while FPFH is a high-dimensional histogram that has a higher level of distinction. However, in RGB-FIPP, global registration is not the end. After this, a local registration will be performed, so the accuracy of RGB-FIPP should be evaluated after local registration. Meanwhile, because local registration is performed with the ICP algorithm, its accuracy depends on that of the ICP algorithm. Currently, the ICP algorithm has been proven to be one of the most accurate methods, so the accuracy of RGB-FIPP will no longer be discussed in this paper.

## 5. Experimental Results and Analysis

In this section, we illustrate the process of point cloud registration, which includes the results of four initial point pairs, global registration with RGB value by using the improved FIPP algorithm, and local registration by using the ICP algorithm. We also discuss the influence of parameters and the comparison of runtime between RGB-FIPP and other methods.

### 5.1. Test Data

**Figure 13:**
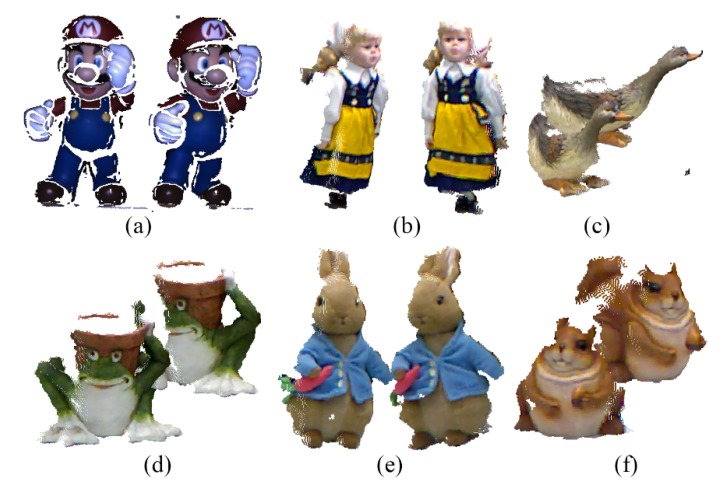
The original datasets: (**a**) SuperMario; (**b**) Doll; (**c**) Duck; (**d**) Frog; (**e**) Peterrabit; (**f**) Squirrel.

To verify the results of the proposed method, in this study we use six sets of point cloud datasets, which name are SuperMario, Doll, Duck, Frog, Peterrabbit and Squirrel. All of datasets were downloaded from the SHOT website (http://www.vision.deis.unibo.it/research/80-shot). Among them, SuperMario datasets were acquired with Space time Stereo and the others with Kinect sensor. Figure 13 shows the six datasets used for the experiment and their original location.

In six sets of datasets, SuperMario have the largest numbers of points which are both more than 40,000. The smallest number is the squirrel datasets which have about 8000 points. The rest of datasets are somewhere in between, about 10,000 to 20,000. The nearest distances between points fall in two types. SuperMario datasets have a nearest distance of 8 cm. Other datasets, which were acquired with Kinect sensor, have the distances of 0.14 to 0.2 cm. The detailed information is shown in Table 2.

### 5.2. Experimental Processes and Results

#### 5.2.1. Color Filtering

Table 3 shows the numbers of original colors and filtered colors for different datasets by using Equation (5). The two SuperMario datasets have 20,485 and 21,306 kinds of RGB values in the target and source datasets, respectively. After color filtering by setting *σ_n_* to 10, the numbers of remaining colors are 593 and 583, which are only about 3% of the original. For other datasets, the values of *σ_n_* are all set to 2. The numbers of filtered colors are between about 2% and 5% of the original color. Among them, the Duck dataset has the most remaining colors and the Squirrel the least. This indicates that the color discriminations of the five types of datasets with Kinect are different. The duck dataset is the most colorful, and the squirrel the least.

The filtered colors and their candidate points will participate in the implementation of the improved FIPP algorithm in order to choose four points pairs. Take the SuperMario datasets as an example, the numbers of the filtered colors are 593 and 583. When the color tolerance is used to generate the candidate points set, 593 colors in target dataset all have candidate points that range from 13 to 119. Therefore, the improved FIPP algorithm will be executed in theses 593 colors and their candidate points.

#### 5.2.2. Selection of Four Points Pairs

**Figure 14:**
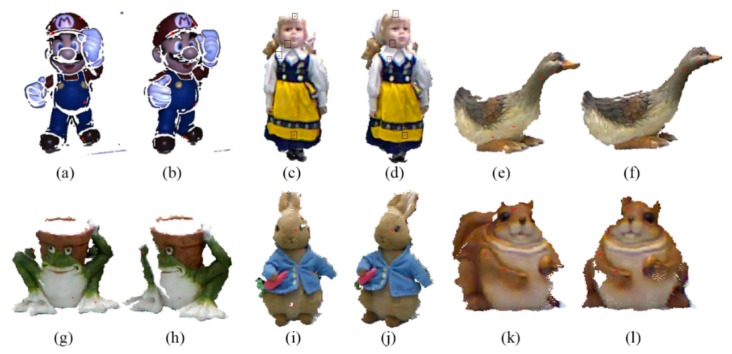
3D RGB information with four initial points: (**a**) Target dataset of SuperMario; (**b**) Source dataset of SuperMario; (**c**) Target dataset of Doll; (**d**) Source dataset of Doll; (**e**) Target dataset of Duck; (**f**) Source dataset of Duck; (**g**) Target dataset of Frog; (**h**) Source dataset of Frog; (**i**) Target dataset of Peterrabit; (**j**) Source dataset of Peterrabit; (**k**) Target dataset of Squirrel; (**l**) Source dataset of Squirrel.

Figure 14 shows the results of searching four point-to-point correspondences in six datasets. The red points are the four points pairs obtained by using the improved FIPP with RGB value, and other colors are the original RGB values.

From Figure 14, the point-to-point correspondences in six datasets are accurate generally, and all four points are scattered on the datasets. This is because that in the improved FIPP algorithm, there is a distance constraint when generating random points and the four points pairs are generated by Equations (1)–(4). Once the four points pairs are generated, the rigid transformation matrix can be solved.

#### 5.2.3. Global Registration

**Figure 15:**
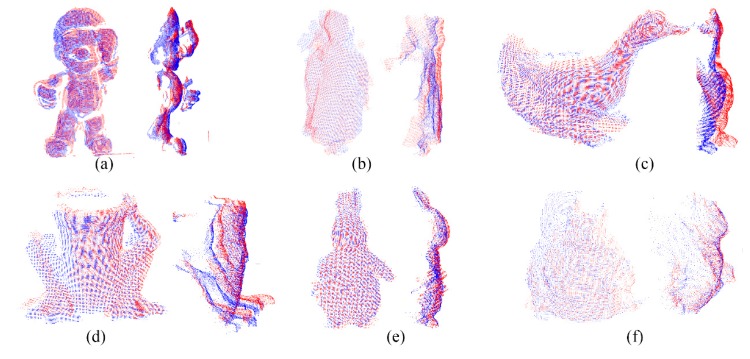
The position of two datasets after global registration with RGB-FIPP algorithm: (**a**) SuperMario; (**b**) Doll; (**c**) Duck; (**d**) Frog; (**e**) Peterrabit; (**f**) Squirrel.

Figure 15 displays the results of global registration, where the red points are the target datasets and the blue are the source. In every sub-figure, the left are the front view and the right are the side view after global registration. The two datasets are all roughly aligned after global registration.

From the result, the precision of registration is relatively low because color, as the descriptor of the point in this article, is not as accurate as other high-dimensional descriptors. However, the method proposed in this paper focuses on the efficiency of registration, and the ICP algorithm will be used to perform the local registration after the global registration. The result of the ICP is the final result, which has been proven to have very high accuracy. The efficiency of the RGB-FIPP will be discussed in Section 5.3 and Section 5.4.

#### 5.2.4. Local Registration

**Figure 16:**
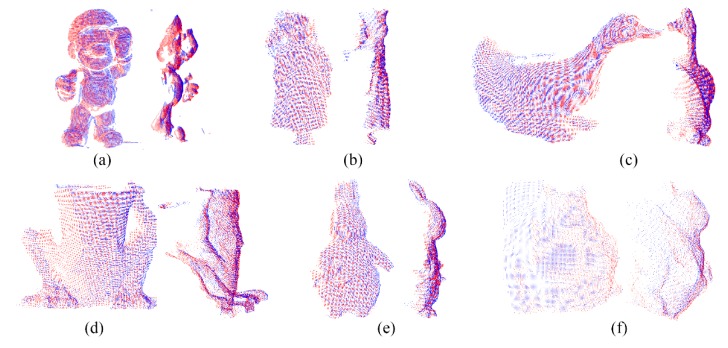
The position of two datasets after local registration with ICP algorithm: (**a**) SuperMario; (**b**) Doll; (**c**) Duck; (**d**) Frog; (**e**) Peterrabit; (**f**) Squirrel.

Figure 16 shows the results of local registration using the ICP algorithm. The position of the two datasets from the front and side views after local registration is shown on the left and right in every sub-figure. After local registration, the two datasets are precisely aligned. In Figure 16, the red and blue points are the target and source datasets, respectively. From the results, the local registration has higher accuracy than the global registration.

#### 5.2.5. Accuracy

As mentioned earlier, the final accuracy of registration is determined by the results of ICP, so only the accuracy of ICP is discussed in this section. In general, RMS is used to evaluate ICP accuracy. However, the nearest distances of the six experimental datasets are different. In order to compare the accuracy of the six datasets uniformly, we use relative RMS for accuracy analysis. Relative RMS is the ratio of RMS to the nearest distance. The formula is as follows:

(11) $$rRMS=\frac{RMS}{d}$$

where r*RMS* is the relative *RMS* and *d* is the nearest distance. Table 4 shows the accuracy of six datasets after the ICP. Except for SuperMario and Squirrel datasets, the relative RMS of the others are 2 to 4 times nearest neighbors. SuperMario has a more than 5 times nearest neighbor, because the datasets have many noises, clutters, occlusions, etc. These points have no point-to-point correspondence in another dataset. The relative RMS of Squirrel is more than 9 times. By observation, the shape of the squirrel is similar to that of a sphere. When the ICP algorithm is used for spherical objects, overlapping two spherical objects as a whole takes precedence over finding the right place. This is the shortcoming of the ICP algorithm.

Overall, the ICP algorithm can achieve high accuracy in point cloud registration [42]. In this paper, the traditional ICP algorithm is used for experiments. In fact, there are many ways to improve the ICP algorithm. If the improved ICP algorithms are used in the method of this paper, the registration accuracy will be higher.

### 5.3. Influence of Parameters

When RGB values are used in global registration, there are two important parameters that may affect the results, i.e., color filter threshold *σ_n_* and color tolerance threshold *σ_c_*. In order to determine the influence of these two parameters on the results, we performed two sets of experiments for both *σ_n_* and *σ_c_*. In the first set of experiments, the values of *σ_n_* were set evenly at a constant interval while keeping *σ_c_* unchanged. In the other set of experiments, the values of *σ_n_* were set to three fixed values and *σ_c_* to regular values from small to large. We choose two datasets with different acquisition techniques for experiments, namely SuperMario (Stereo) and Doll (Kinect). The experiments were performed 10 times in succession for each parameter value and the runtime was recorded for comparison.

#### 5.3.1. Color Filter

**Figure 17:**
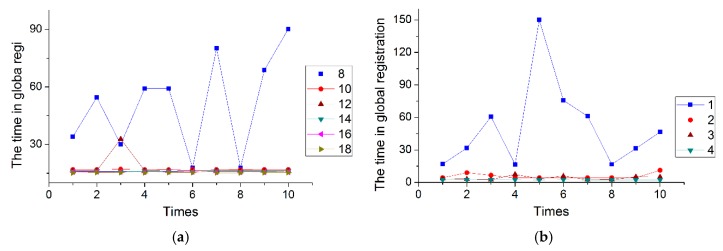
The time with the different filtering parameters *σ_n_*: (**a**) SuperMario; (**b**) Doll.

In this set of experiments, *σ_n_* is evenly set from 8 to 20 at intervals of 2 for Stereo and from 1 to 4 at an interval of 1 for Kinect, while keeping *σ_c_* constant. Figure 17 shows the results for Stereo and Kinect, and different numbers in the legend represent different values of *σ_n_*.

From Figure 17, it is obvious that runtime decreases as *σ_n_* increases for both Stereo and Kinect, and when *σ_n_* is small, the runtime is very long, while it will be reduced quickly and begins to stabilize as *σ_n_* is added to a certain value. From the difference, we can see that the runtime for Stereo is higher than that of Kinect results from the distinction of colors in Stereo being less than that in Kinect, and because the Stereo dataset has a significant amount of occlusion, clutter, and outliers. In addition, when *σ_n_* is greater than 10, the runtime for Stereo tends to be stable, while for a stable Kinect runtime this value is 2. This occurs because the number of points in Stereo is greater than in Kinect, and thus a larger value of *σ_n_* should be set to filter out more points to stabilize the runtime. It is important to note that larger values of *σ_n_* are not better, because when *σ_n_* is too large the number of remaining colors after filtering may be too small to generate four initial point pairs. For example, if *σ_n_* is more than 4 in the Kinect datasets, four initial point pairs cannot be found.

#### 5.3.2. Color Tolerance

**Figure 18:**
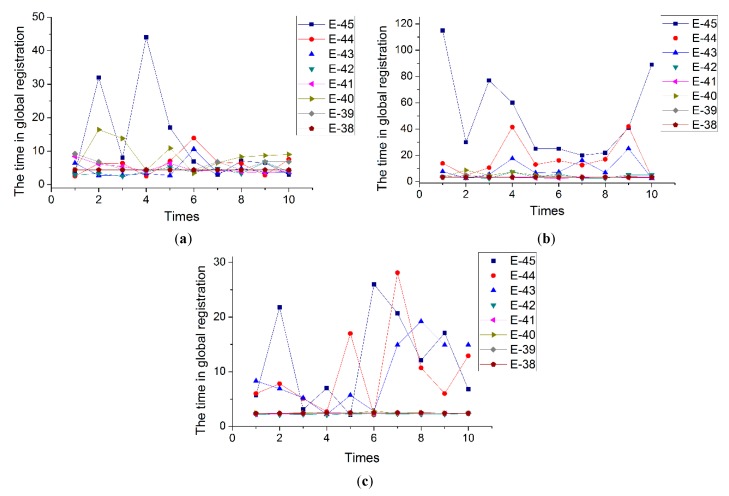
The time with the different color tolerance in Doll dataset: (**a**) *σ_n_* = 2; (**b**) *σ_n_* = 3; (**c**) *σ_n_* = 4.

In another set of experiments, the color tolerance *σ_c_* was set to regular values, while *σ_n_* was set to three different values. The principle of setting values of *σ_n_* is based on the runtime being stable in the first set of experiments. Here, *σ_n_* was set to 10, 12, and 14 for Stereo and to 2, 3, and 4 for Kinect. Additionally, in this paper, the type of original point cloud datasets used in the experiments is floating-point and the values were saved with the power of E, so the values of *σ_c_* were set according to the power series of E, ranging from E-46 to E-38. Similarly, if the type of data is integer denoted by R, G, and B, *σ_c_* can be evenly set to a constant interval. Table 5, Table 6 and Table 7 indicate the runtimes of 10 experiments, and the average time and standard deviation in the Stereo datasets when the *σ_n_* values are 10, 12, and 14, respectively. Figure 18 display the runtimes of 10 experiments in the Kinect datasets when the *σ_n_* values are 2, 3, and 4, respectively.

We can see from Table 5, Table 6 and Table 7 that the difference in runtime for the Stereo datasets, most of which is between 15 and 20 s, is not significant with different values of both *σ_n_* and *σ_c_*. However, there are some nuances worth mentioning. When *σ_c_* increases, although some runtimes increase and some decrease, the overall trend of runtime is a decrease and runtime is the lowest with maximum *σ_c_*. Furthermore, it is slightly obvious that the standard deviation decreases as *σ_c_* increases. This indicates that the larger the value of *σ_c_*, the more stable the global registration.

Figure 18 indicate the trend of runtime with *σ_n_* and *σ_c_* values in the Kinect datasets, and the regularity is stronger than in the Stereo datasets. As *σ_c_* increases, runtime generally decreases. Moreover, when *σ_c_* is small, runtime is very different; that is, the maximum can reach tens of seconds or even more than 100 s while the minimum is less than 5 s. However, runtime is gradually stabilized with *σ_c_*, and once stabilized the runtimes are almost the same. In addition, with increasing of *σ_n_*, convergence speed becomes increasingly faster.

Comparing the results of the two sets of experiments, likewise, the stability of runtimes all increased with increasing *σ_c_*. The difference is that the runtime in Kinect will drop and eventually stabilize with *σ_c_*, while the change in Stereo is not significant. Considering that the color discrimination of Kinect datasets is greater than that of Stereo, while the number of points is less, the number of candidate points to a given point is also less; therefore, more time is required to search point-to-point correspondence. Furthermore, if *σ_c_* is increased to a certain size, the number of candidate points will be sufficient for searching point-to-point correspondence, and thus the time will decrease. It is also important to note that a larger value of *σ_c_* is not better either, because larger values of *σ_n_* result in greater registration.

### 5.4. Efficiency Comparison

#### 5.4.1. Comparison with Descriptors

**Figure 19:**
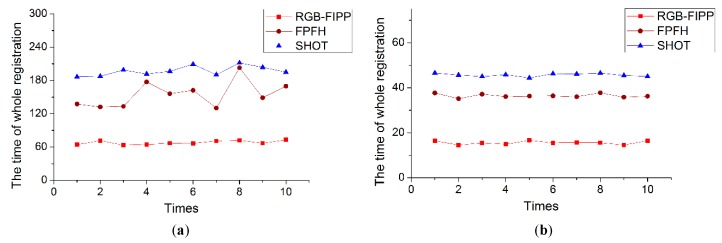
The time of whole registration: (**a**) SuperMario; (**b**) Doll.

In order to verity the efficiency of method proposed in this paper, we compare the efficiency of RGB-FIPP with FPFH and SHOT, because FPFH and SHOT achieve the best computational performance [24]. We used the same computer to execute the three methods without any accelerated processing of the programs. At the same time, the parameters of FIPP were the same in all methods, except for the improvement described in this paper, and *σ_n_* values were set to 10 and 4 in the Stereo and Kinect datasets, respectively and the *σ_c_* values were both set to E-42. Because the ICP algorithm is used to perform local registration after global registration, which can achieve high accuracy, only the computational efficiency of the three methods is used for comparison. Experiments were done in six groups, three of which were used to compare SuperMario datasets and three for Doll. Figure 19 indicate the results for Stereo and Kinect, respectively.

As can be seen from Figure 19, the runtimes of using FPFH and SHOT are both higher than RGB-FIPP for the two datasets. In the SuperMario experiments, the runtime of FPFH ranges from 130 to 210 s, and the runtime of SHOT from 186 to 209 s. Overall, the average time of FPFH is 155 s, which is better than SHOT’s 197 s. However, the runtime of RGB ranges from 53 to 63 s, which is much smaller than FPFH and SHOT. For Doll datasets, the runtime of FPFH ranges from 35 to 38 s and that of SHOT ranges from 46 to 49 s. The time of RGB ranges from 15 to 17 s. Similarly, the results of RGB-FIPP are better than FPFH and FPFH are better than SHOT. In addition, when using SuperMario datasets, FPFH and SHOT are unstable. This is because the SuperMario datasets have a lot of occlusion and dispersion. RGB-FIPP results are very stable in both datasets. These results illustrate that the proposed method is much faster and more stable than FPFH.

Moreover, to understand the differences in detail, the registration process was split into several parts. In the RGB-FIPP method, the process includes RGB value statistics (including color filtering, RGB tolerance, and candidate points set), searching point-to-point correspondence, and ICP registration. In the method FPFH and SHOT, the process includes calculation of FPFH and searching point-to-point correspondence. Table 8 and Table 9 present the comparisons of the two datasets.

As can be seen from Table 8 and Table 9, the calculation times for three descriptors are all very stable as a whole, however, the times of calculating RGB value are much less than the other two. The runtimes of searching point-to-point correspondence using FPFH and SHOT in SuperMario datasets are very unstable, ranging from 11 to 81 s and from 8 to 31 s, respectively. In addition, the runtimes of searching point-to-point correspondence with RGB value are all far less than FPFH and SHOT, because RGB-FIPP only searches for four points pairs. It is worth mentioning that this step of RGB-FIPP is less accurate than the other two methods, but it can be compensated by the subsequent ICP algorithm. Even if the time for ICP is added, the total time by RGB is still less than the others. The average total time of the RGB-FIPP method is 15.5 s, which is better than 36.4 s of FPFH and 48.2 s of SHOT. Thus, 3D point cloud registration using the method proposed in this paper can achieve both faster speed and high accuracy.

In addition to the real runtime comparison of RGB-FIPP with FPFH and SHOT, Table 10 shows the comparison of RGB and main descriptors in terms of computational complexity and dimensions, including Spin Image (SI), 3D Shape Context (3DSC), Unique Shape Context (USC), Rotational Projection Statistics (RoPS), Tri-Spin-Image (TriSI), Local Surface Patch (LSP), Point Feature Histogram (PFH), FPFH, Signature of Histogram of Orientations (SHOT).

In terms of computational complexity, PFH has the highest complexity which is *O*(*k*^2^*n*). RoPS and TriSI are both *O(*3*kn*) because they are counted separately in three directions. All other descriptors are *O*(*kn*), except RGB-FIPP is *O*(*n*). In terms of the dimensions represented by the value of the descriptor, 3DSC and USC have the highest dimensions, which are both 1980. Besides RGB-FIPP, FPFH has the lowest dimension at 33. The other descriptors have dimensional values in between. It can be seen that the computational complexity of the common descriptors is optimally *O*(*kn*), and the dimension of the value ranges from tens to hundreds. However, the complexity of RGB-FIPP is *O*(*n*) and the dimension is 3. Therefore, the computational complexity of RGB-FIPP is the lowest, and it is the fastest.

#### 5.4.2. Comparison with Other Registration Algorithms

Table 11 shows a comparison of computational complexity between RGB-FIPP and other registration algorithms.

A RANSAC-based method is used to find three points in a target dataset and its corresponding points in a source dataset by traversing all points, in order to find the best transformation. The computational complexity of RANSAC is *n*^3^, and it is only usable with a small number of datasets. RANSAM reduces the number of traversed points to two, and transformation is determined by two points and their normal, so its complexity is *n*^2^. GIA extracts feature points with an integral invariants volume (IID) descriptor and looks for correspondences using a branch-and-bound algorithm, so the complexity is $kn+C^{2\cdot }{}^{\ln_{2}{}^{m}}$, where *k* is the number of neighbor points, and *C* and *m* are the number of candidate and feature points, respectively. Although 4PCS must find four points, three of the four are close to each other, so the complexity is *n*^2^. EC is a searching strategy based on computational models of evolutionary processes, in which the complexity with *n^k^* is determined by the number of evolutions, where *k* is usually greater than 2. The complexity of FIPP and RGB-FIPP is *kn* + *k’C*^4^ and *n* + *k’C*^4^, respectively, where *k’* is the times of finding four initial point pairs, and *C* is the number of candidate points. Since there is no need to consider neighborhoods, the complexity of RGB-FIPP is the lowest, followed by FIPP and GIA due to the very small value of *C*, and then RANSAM and 4PCS, with RANSAC and EC being the highest.

In terms of accuracy, there is no proper experimental comparison for all the methods. Diez [43] compared the 4PCS and RANSAC methods with huge point clouds, and indicated that 4PCS is more accurate than any other RANSAC-based method. Santamaria [29] proved the accuracy of EC in outperforming the ICP algorithm, but its computational times are very expensive. However, the result of global registration is not the end, and subsequent implementation of local registration is still required. Even if the accuracy of global registration is slightly lower, the ICP algorithm used in local registration can achieve a high accuracy because a threshold that can satisfy the precision requirement is used to control whether the iteration is terminated. Therefore, the RGB-FIPP method is not only very fast, but can also meet the accuracy requirement.

## 6. Discussion

The determination of candidate point set is based on the RGB value, and parameters of color filter *σ_n_* and color tolerance *σ_c_* both have an important effect on global registration. Parameter *σ_n_* determines the number of colors involved in searching point-to-point correspondence. Generally, the larger the value of *σ_n_*, the less the number of colors. Although the number is getting smaller with *σ_n_*, the average number of points with the same color is more, which increases the possibility of the same points with the same color from two datasets and improves registration efficiency. However, there is an upper limit for *σ_n_*. Once the value of *σ_n_* is more than a specific value registration will fail because the number of the same points with the same color is not enough to generate a rigid transformation matrix. Therefore, when the runtime drops to a lower value and will not change much anymore with the increase of *σ_n_*, the value of *σ_n_* is the best, for example 10 in Stereo and 4 in Kinect.

Parameter *σ_c_* determines the number of candidate points with the same color. The larger the value of *σ_c_*, the greater the likelihood of the same points. Nevertheless, the specific impact on global registration depends on the distinction of color. If the distinction is small, the number of candidate points with the same color is so much that can find enough point pairs to registration without a larger value of *σ_c_*. In this situation, the value of *σ_c_* has little effect on run time, and vice versa, that can be seen in Table 5, Table 6, Table 7 and Figure 18. Moreover, despite the influence of *σ_c_* depends on the distinction of color of the point cloud, there have the same rule that stability of registration is getting higher and higher with the increase of *σ_c_*. However, the increase in stability of runtime also sacrifices the accuracy. So, the choice of the value of *σ_c_* should follow the principle of balance between runtime and accuracy. And here, the values are both set to E-42.

Moreover, FIPP was improved for searching point-to-point correspondence with RGB value. The improved FIPP can reduce the time of searching correspondence. Although the registration accuracy is sacrificed because it is rough to regard RGB as the descriptor, fortunately this defect can be compensated by local registration using the ICP algorithm.

Overall, the RGB-FIPP method for 3D point cloud registration proposed in this paper has a very fast speed and high accuracy. It should be noted that this method requires that 3D point cloud data have RGB values, while the distinction of color has a great influence on the operational efficiency of the algorithm. If the 3D point cloud data do not have RGB values, RGB-FIPP cannot be used for point cloud registration.

## 7. Conclusions

In this paper, we presented a fast method for 3D point cloud registration by using RGB value of the point, which is called RGB-FIPP. Color information instead of traditional descriptors to express the characteristics of points and it reduces the computational complexity of computing descriptor from *O*(*kn*) to *O*(*n*). And then, an improved FIPP algorithm is used to search point-to-point correspondences for global registration. Finally, the ICP algorithm is used to perform the local registration. Using color value of the points greatly improves the efficiency of the entire registration, meanwhile, the ICP algorithm ensures the final accuracy. On the whole, the method in this paper improves efficiency while ensuring accuracy. For future work, we plan to combine detectors or descriptors with RGB values in registration in order to improve the descriptiveness of a 3D point.

## Figures and Tables

**Table 1:** The distribution of colors with the same number of points in two datasets.

The Number of Points in the Same Color	The Number of Colors with the Same Number of Points in Target Dataset	The Ratio of Colors with the Same Number of Points in Target Dataset	The Number of Colors with the Same Number of Points in Source Dataset	The Ratio of Colors with the Same Number of Points in Source Dataset
1	15,772	79.34%	17,100	78.38%
2	1946	9.79%	2268	10.40%
3	621	3.12%	751	3.44%
4	290	1.46%	395	1.81%
5	196	0.99%	215	0.99%
6	146	0.73%	149	0.68%
7	115	0.58%	106	0.49%
8	76	0.38%	102	0.47%
9	57	0.29%	75	0.34%
10	68	0.34%	74	0.34%
10 < *n* ≤ 20	317	1.59%	338	1.55%
20 < *n* ≤ 30	137	0.69%	117	0.54%
*n* > 30	139	0.70%	128	0.59%

**Table 2:** The comparison of six datasets.

Dataset	Sensor	Points Number of Target Dataset	Points Number of Source Dataset	Nearest Neighbor
SuperMario	Stereo	41,702	44,276	8 cm
Doll	Kinect	13,609	14,083	0.2 cm
Duck	Kinect	25,109	26,905	0.15 cm
Frog	Kinect	26,623	23,656	0.15 cm
Peterrabbit	Kinect	13,944	13,357	0.15 cm
Squirrel	Kinect	8353	7331	0.14 cm

**Table 3:** The Comparison of color before and after filtering of six datasets.

Dataset	Color Number		Number of Filtered Colors	
	Target Dataset	Source Dataset	Target Dataset (%)	Source Dataset (%)
SuperMario	20,485	21,306	593 (2.89%)	583 (2.74%)
Doll	9950	10,515	415 (4.17%)	420 (3.99%)
Duck	18,277	19,810	966 (5.29%)	970 (4.90%)
Frog	18,160	16,311	648 (3.57%)	510 (3.13%)
Peterrabbit	10,052	10,319	388 (3.86%)	486 (4.71%)
Squirrel	5716	6391	142 (2.48%)	197 (3.08%)

**Table 4:** Accuracy analysis of six experimental datasets.

Dataset	RMS (m)	Nearest Neighbor (cm)	Relative RMS
SuperMario	0.4422	8	5.5275
Doll	0.0049	0.2	2.4500
Duck	0.0058	0.15	3.8667
Frog	0.0041	0.15	2.7333
Peterrabbit	0.0039	0.15	2.6000
Squirrel	0.0129	0.14	9.2143

**Table 5:** The time with the different color tolerance when *σ_n_* is 10 in Stereo datasets.

Serial Number of Experiments	E-46	E-45	E-44	E-43	E-42	E-41	E-40	E-39	E-38
1th Time	18.7	18.1	19.0	19.9	18.7	18.7	16.8	16.8	16.7
2th Time	16.9	14.4	17.3	16.7	17.5	20.3	17.0	16.9	16.9
3th time	27.2	15.8	18.7	18.7	17.2	20.6	17.3	16.9	16.9
4th Time	15.7	20.9	17.3	17.2	18.7	16.9	17.0	16.9	16.8
5th Time	15.0	16.9	18.2	19.7	17.4	17.2	17.4	20.8	16.7
6th Time	20.8	21.6	17.3	17.7	19.1	17.0	17.1	16.8	16.9
7th Time	15.1	19.1	17.0	18.4	17.6	17.1	16.7	16.9	16.7
8th Time	15.2	22.5	20.0	19.3	17.6	17.2	22.1	20.3	16.8
9th Time	15.0	16.0	18.6	18.4	17.5	17.5	21.3	16.8	16.8
10th Time	16.4	16.3	18.4	17.5	19.5	17.5	17.8	16.9	16.8
Average	17.60	18.16	18.18	18.18	18.08	18.00	18.05	17.60	16.80
Standard deviation	3.67	2.62	0.91	1.02	0.79	1.32	1.86	1.48	0.08

**Table 6:** The time with the different color tolerance when *σ_n_* is 12 in Stereo datasets.

Serial Number of Experiments	E-46	E-45	E-44	E-43	E-42	E-41	E-40	E-39	E-38
1th Time	18.2	16.7	17.8	20.7	16.5	17.1	17.2	16.4	16.5
2th Time	17.9	18.1	17.4	21.3	16.6	16.5	17.1	17.3	16.3
3th time	15.2	14.9	17.9	18.4	17.0	16.6	16.5	16.4	16.3
4th Time	16.5	17.2	15.9	19.0	16.3	16.6	17.3	16.4	16.3
5th Time	19.7	25.6	17.2	20.6	18.0	16.6	16.8	16.3	16.6
6th Time	18.1	16.1	21.3	16.0	16.4	18.0	16.9	16.4	16.4
7th Time	17.0	21.9	18.8	16.9	15.9	16.8	17.2	16.5	16.3
8th Time	18.3	14.5	16.7	17.3	17.1	16.9	16.3	16.3	16.3
9th Time	19.9	16.1	16.4	19.5	19.0	17.2	17.5	16.3	16.4
10th Time	14.8	20.4	19.6	19.5	16.3	16.9	16.8	16.2	16.5
Average	17.56	18.15	17.90	18.92	16.91	16.92	16.96	16.45	16.39
Standard deviation	1.61	3.32	1.54	1.67	0.89	0.42	0.35	0.29	0.10

**Table 7:** The time with the different color tolerance when *σ_n_* is 14 in Stereo datasets.

Serial Number of Experiments	E-46	E-45	E-44	E-43	E-42	E-41	E-40	E-39	E-38
1th Time	25.4	14.6	18.0	22.3	17.1	18.3	16.8	18.7	15.9
2th Time	14.3	15.8	19.3	22.0	17.1	16.9	16.8	18.8	16.0
3th time	21.0	14.4	17.2	16.3	16.3	18.3	16.1	16.2	15.9
4th Time	16.7	17.8	21.8	18.4	19.2	17.1	16.1	16.1	16.0
5th Time	15.7	14.3	16.4	18.8	19.1	16.9	16.7	16.1	16.0
6th Time	14.4	18.1	19.8	17.5	20.4	17.1	16.8	18.5	15.8
7th Time	15.5	24.8	19.3	20.2	16.9	17.3	17.7	16.2	16.0
8th Time	22.2	16.9	18.4	17.1	17.4	18.4	17.0	16.1	15.9
9th Time	15.1	18.4	17.3	17.0	16.7	16.7	17.2	16.1	15.9
10th Time	15.3	18.0	17.5	26.7	18.1	17.1	16.8	16.1	16.1
Average	17.56	17.31	18.50	19.63	17.83	17.41	16.80	16.89	15.95
Standard deviation	3.67	2.93	1.51	3.07	1.26	0.62	0.45	1.17	0.08

**Table 8:** Comparison of run time in SuperMario datasets.

Experiment Number	RGB-FIPP			FPFH		SHOT	
	RGB Value	Points Pairs	ICP	FPFH Value	Point Pairs	SHOT Value	Point Pairs
1th time	13.1	3.3	48	120	17.54	172	14.32
2th time	13.1	3.3	55	121	11.28	178	8.56
3th time	13.1	3.3	47	121	12.26	183	15.36
4th time	13.3	3.3	48	121	56.31	175	16.21
5th time	13.6	3.4	50	120	36.10	171	24.56
6th time	13.4	3.3	50	121	41.23	176	32.91
7th time	13.5	3.4	54	121	9.25	176	13.40
8th time	13.6	3.4	55	121	81.89	174	37.56
9th time	13.6	3.4	50	121	27.78	175	27.78
10th time	13.6	3.4	56	121	48.61	176	18.61
Average time	13.39	3.35	51.3	120.8	34.225	176	20.927

**Table 9:** Comparison of run time in Doll datasets.

Experiment Number	RGB-FIPP			FPFH		SHOT	
	RGB Value	Points Pairs	ICP	FPFH Value	Point Pairs	SHOT Value	Point Pairs
1th time	1.98	0.47	14	34	3.69	46.58	2.10
2th time	1.99	0.51	12	33	2.13	45.65	2.38
3th time	1.99	0.5	13	34	3.15	44.99	2.91
4th time	1.97	0.93	12	34	2.06	45.77	1.89
5th time	1.99	0.65	14	34	2.29	44.41	3.01
6th time	1.99	0.53	13	33	3.39	46.25	2.59
7th time	1.99	0.6	13	34	2.03	46.09	3.20
8th time	1.97	0.58	13	34	3.75	46.51	3.18
9th time	1.99	0.55	12	34	1.77	45.48	1.95
10th time	1.99	0.49	14	33	3.21	45.04	2.04
Average time	1.985	0.581	13	33.7	2.747	45.68	2.53

**Table 10:** Comparison of computational complexity of descriptors.

Descriptor	Computational Complexity	Dimension
SI	*O*(*kn*)	*225*(*d*^2^)
3DSC	*O*(*kn*)	*1980*(*d*^3^)
LSP	*O*(*kn*)	578(*d*^2^)
USC	*O*(*kn*)	1980(*d*^3^)
RoPS	*O*(3*kn*)	135(5 *d*^3^)
TriSI	*O*(3*kn*)	675(3 *d*^2^)
PFH	*O*(*k*^2^*n*)	125(*d*^3^)
FPFH	*O*(*kn*)	33(3*d*)
SHOT	*O*(*kn*)	352(*d*^4^)
RGB-FIPP	*O*(*n*)	3

**Table 11:** Comparison computational complexity between different registration algorithms.

	Computational Complexity	Descriptor
RANSAC	*n* ^3^	N
RANSAM	*n* ^2^	N
GIA	$kn+C^{2\cdot }{}^{\ln2^{m}}$	Y
4PCS	*n* ^2^	N
EC	*n^k^*	N
FIPP	*kn* + *k’C*^4^	Y
RGB-FIPP	*n* + *k’C*^4^	Y
